# Characteristics and Determinants of Patients Discontinuation of Breast Cancer Follow-Up Care at the Radiation Oncology Department, University College Hospital, Ibadan, Nigeria

**DOI:** 10.1155/2018/1597964

**Published:** 2018-08-12

**Authors:** M. D. Dairo, D. B. Adamu, Y. A. Onimode, A. Ntekim, O. Ayeni

**Affiliations:** ^1^Department of Epidemiology and Medical Statistics, Faculty of Public Health, College of Medicine, University of Ibadan, Nigeria; ^2^Department of Radiation Oncology, University College Hospital Ibadan, Nigeria; ^3^Department of Radiology, Gombe State University, Nigeria; ^4^Nuclear Unit, Department of Radiation Oncology, College of Medicine, University of Ibadan, Nigeria

## Abstract

**Objectives:**

The aim of this study is to describe the characteristics and predictors of discontinuation during follow-up care among breast cancer patients at the Radiation Oncology Department, University College Hospital (UCH), Ibadan, Nigeria.

**Methodology:**

This is a retrospective cross-sectional study of 504 patients with histological diagnosis of breast cancer referred for radiotherapy to the breast or chest wall. Data extraction form was used to obtain information on sociodemographic and disease related variables and time to discontinuation of care. Discontinuation rates and its predictors were estimated using Kaplan-Meier, Log rank test, and Cox's regression method of analyses.

**Results:**

Five hundred and four breast cancer patients were studied. The mean age was 47.7years, 58.2% presented late with advanced stage disease, and 40% and 39% had metastasis and anaemia, respectively. Seventy-seven percent of patients discontinued follow-up care before completion of ten-year period. The 5-year and 10-year discontinuation rates were 69.8% and 92.6%, respectively. The median discontinuation time was 44 months. Discontinuers were more likely to be older than the age of 45years {HR=1.415; 95% CI= 1.044 - 1.917}, have metastasis {HR=1.793; 95% CI=1.396 - 2.302}, be anaemic {HR=1.404; 95% CI = 1.120 - 1.760)}, and have late-stage disease {HR=1.310; 95% CI = 1.407-1.639)}.

**Conclusion:**

Breast cancer care discontinuation is associated with late presentation and advanced stage of disease. Therefore a system of community follow-up care and public awareness about breast cancer symptoms is recommended to reduce late presentation and discontinuity of care.

## 1. Introduction

Breast cancer is the leading female malignancy in the world and is now the most common cancer in Nigeria [1]. The peak incidence of the disease in Nigeria is at least a decade earlier compared to the Caucasians [2]. The incidence of breast cancer in Nigeria is on the increase from 13.8 to 15.3 per 100,000 in 1992 to 116 per 100,000 in 2001 in Ibadan [3].

The major constraint in the management of breast cancer in Nigeria is the limitation of resources because patients bear the burden of paying for cancer treatment. Having a population of over 180 million and a Gross Domestic Product of about 2000US Dollar per capital annually, Nigeria ranks among the poorest nations in the world. The National Health Insurance Scheme is still in its formative stage; thus payment for cancer treatment is mostly out of pocket. Thus a significant proportion of patients do not present for treatment, may not complete the prescribed courses of treatment, and do not attend posttherapy surveillance, the follow-up care required to maintain better health status, assess effectiveness of therapy, and detect and treat early recurrence of the disease [4]. Recent study in Ibadan reported that majority of breast cancer recurrences were detected within 2 years of primary treatment, indicating the necessity of follow-up evaluations [5].

Loss to follow-up is a major challenge in the successful management of breast cancer patients in Nigeria and sub-Saharan Africa; true outcomes of patients lost to follow-up thus become difficult to assess. In Nigeria, as indeed in many developing countries, a combination of poor education, poverty, and a high percentage of nonorthodox healing practices among the populace contribute to late presentation of breast cancer in many hospitals with consequent high occurrence of metastatic disease and poor disease survival [6]. This is worsened by the commonly encountered nonadherence to treatment schedule among the patients. The burden of caring for these large numbers of patients in a low resource country is enormous.

There are five functional radiotherapy centres in Nigeria today with a population of over 150million. This gives a ratio of one radiotherapy centre to about 30million persons, a far cry from the WHO recommendation of 1: 250,000 persons [7]. Thus patients will have to travel long distances for radiotherapy. Longer distances imply more financial burden on the patient's caregivers; the family members who have to take more time off from work suffer loss of pay and incur the costs of feeding, travelling, and accommodation at the referral hospital. Such expenditure can be sufficiently enormous to discourage the patient from adherence to routine follow-up care. The benefits of follow-up in breast cancer patients include early detection of potentially curable events, management of therapy related side effects, psychosocial care, support and counselling, encouragement and support for physical exercise, and weight reduction during follow-up in order to improve quality of life and physical performance, reevaluation of current adjuvant therapy, and monitoring of compliance with endocrine therapies. However, these benefits are missed by patients discontinuing follow-up.

Most studies on breast cancer in Nigeria emphasized pathology, epidemiology, and clinical features of the disease with few attempts at determining survival among these patients [1, 3, 5, 8–11]. It is essential to target this group of patients and understand the sociodemographic and disease related barriers to posttherapy follow-up in order to provide strategic interventions to prevent loss to follow-up and a better patient outcome. The aim of this study therefore was to define the characteristics of breast cancer patients and identify determinants or predictors of discontinuation of follow-up care among them.

## 2. Methodology

### 2.1. Study Setting

The study was carried out at the Radiotherapy Clinic, University College Hospital (UCH), Ibadan, Nigeria. Radiotherapy department was established in the year 1987. It is equipped with a Telecobalt and HDR Co60 Brachytherapy machines with modern treatment planning system. The department has 6 Radiation Oncologists and 2 certified medical Physicists. It serves as a referral centre for cancer patients that require radiation therapy from units within UCH and clinics and hospitals within and outside Ibadan (patients from all over the country including some West African countries). Annually the department treats about 200-300 breast cancer patients. The study population consists of patients with histological diagnosis of breast cancer referred for radiotherapy to the breast or chest wall.

### 2.2. Study Design

This is a retrospective cross-sectional study on breast cancer patient's follow-up. The study participants included breast cancer patients with histological diagnosis referred for adjuvant radiotherapy of the breast or chest wall at the Radiotherapy Department, UCH, Ibadan, over a period of ten years. The records of the following categories of patients were excluded from the study: breast cancer cases without accompanying histological confirmation of diagnosis and breast cancer cases without case file or radiotherapy treatment cards and without at least one follow-up record.

### 2.3. Data Collection Instrument and Procedures

Hospital case files with follow-up records and radiotherapy treatment cards of breast cancer patients attended to between 2001 and 2010 were retrieved from the radiotherapy clinic medical records by the investigators. The data was collected using a data extraction form which consist of age at diagnosis, sex, educational status, marital status, menopausal status, parity, stage of breast cancer at diagnosis (Manchester staging classification was used as it is the commonest staging classification used by surgeons referring patients for radiotherapy outside of Ibadan), grade of differentiation, histology of breast cancer, lymph node status, development of metastasis, duration of symptoms before diagnosis, distance of home town from University College Hospital, Ibadan (in kilometers), surgery performed (no surgery, breast conservation surgery or radical surgery), whether patient received adjuvant chemotherapy or radiotherapy to the chest wall, baseline packed cell volume (PCV), and status of patient from diagnosis to discontinuation of follow-up. The status was either loss to follow-up or censored (survival beyond end of study or death) and time to event, that is, loss to follow-up in months.

### 2.4. Data Management and Analysis

The data were carefully entered and analyzed using SPSS version 16.0. Regular checks were done to detect and correct errors. The dependent or the outcome variable was defined as the time from histological diagnosis to discontinuation of follow-up care. Discontinuation of care is defined as missing three consecutive follow-up appointments. The independent variables explored are the sociodemographic characteristics and clinical factors such as age, distance of home town from the University College Hospital, marital status, duration of disease symptoms before diagnosis, educational status, stage of disease, baseline PCV, and metastasis. Frequency, percentage, mean, median, range, and standard deviation were used to summarize the sociodemographic, clinical, and treatment variables of the patients and presented using tables and graphs. Discontinuation curves were plotted using Kaplan-Meier method and curves were compared using Log rank test. Log rank test was used to test for an association between dependent variable (loss to follow-up) and independent variables such as age, marital status, educational status, menopausal status, duration of symptom before diagnosis, distance of home/town to UCH, Ibadan, stage of disease at presentation, lymph node status, histological grade, metastasis, and anaemia. The variables were considered to show significant association when the p value was less than 0.05. The discontinuation of patients with breast cancer was compared using Log rank test according clinical features. The clinical features were dichotomized for ease of comparison between the clinical subgroups. Multivariate analysis was carried out using Cox-proportional hazard models to determine the predictors of discontinuation of follow-up. This was done using covariates that showed statistically significant association with follow-up discontinuation at p < 0.1 on bivariate analysis. In analysis of discontinuation using Kaplan-Meier, time of origin was taken as the time of breast cancer diagnosis. The patient status at 31st December, 2010, was categorized as alive, dead, and lost to follow-up. The endpoint of patient was follow-up discontinuation. Patient that died before or found alive at end of December 2010 was censored. The median discontinuation times were obtained from the Kaplan-Meier discontinuation curve.

Multivariate Cox's proportional hazard model is expressed as follows:

(1) $$\begin{array}{c} h\left(\;t,X\;\right)=h_{0}\left(\;t\;\right)\exp\left(\;\overset{p}{\underset{i=1}{\sum }}\beta_{i}x_{i}\;\right) \end{array}$$

where

 
*X* = (*x*_1_, *x*_2_, ⋯, *x*_*p*_) is explanatory/predictor variables;
 
*h*_0_(*t*) is baseline function (the hazard function for an individual for whom all the values of the independent variables are zero);
 
*β* is the vector of coefficients of the independent variables *X*_*i*_.

The general Cox's proportional hazard model was fitted for the following: age, marital status, stage of disease, PCV status, duration of symptoms, distance of home/town from Ibadan, and metastasis. They were statistically significant in the Log rank test.

The proportional hazard model now becomes

(2) ht=h0texp⁡β1Age+β2Anaemia+β3stage  of  disease+β4Metastasis+β5Marital  status+β6Menopausal  status+β7Duration  of  symptoms+β8Distance  of  home  from  Ibadan

### 2.5. Ethical Consideration

Ethical clearance to conduct the study was obtained from the joint Ethical Review committee of the University of Ibadan/University College Hospital, Ibadan. All information collected in this study were coded with numbers and hospital numbers and the names of patients were not used to maintain confidentiality. The data extraction forms were kept in a locked cupboard; the data entered on the computer were password protected and are accessible to the researcher only. During the study, there was no contact between the patients and the researcher. The study was noninvasive and without any harm to the patients.

## 3. Results

### 3.1. Patients Sociodemographic Characteristics

A total of 504 (Females: n=500, Males: n=4) patients satisfied the inclusion criteria.  Table 1 showed that the mean age of patients was 47.69 years with a standard deviation of 10.63. Most of the patients are between the ages of 41 and 50 years (34.1%); the mean age is 61.25% for males and 47.58% for females, respectively. The majority of women with breast cancer were postmenopausal 272 (54.0%), while 216 (42.9%) were premenopausal and 16 (3.2%) had unknown menopausal status.

### 3.2. Patients Clinical Characteristics

The commonest site of breast cancer was on the left breast [244, (48.4%)] while the right breast accounted for about 227 (45.0%) and 33 (6.5%) of breast cancer was seen in both breasts during the study. Stages I, II, III, and IV accounted for 29 (5.8%), 177 (35.1%), 138(27.4%), and 155 (30.8%), respectively. Early-stage breast cancer (stages I&II) accounted for 40.9% while late-stage breast cancer (stages III&IV) constituted the majority. Most of patients (86.5%) had positive auxiliary nodes; ipsilateral axilla was seen in 84 (16.7%). Status of axillary nodes could not be determined in 24 (4.8%) of patients.

Invasive ductal carcinoma [443, (87.9%)] accounted for the most common histological type of breast cancer seen. Grade II breast cancer was also the most common, being seen in 303 (60.1%) of the cases, while 202(40.1%) had distant metastases. The metastasis was more commonly found in the bone (116, 23.0%) and the lungs [104, (20.6%). Three hundred and ten patients (61.5%) had a normal PCV ≥ 33%) at baseline before treatment while 194 (38.5%) had low packed cell volume (PCV <33%). Most of the women with breast cancer underwent radical breast surgery [413, (81.9%)]. Almost all the patients completed chemotherapy and radiotherapy but more than half [268, (53.2%)] did not complete hormonal therapy (Table 2).

### 3.3. Factors Associated with Discontinuation of Follow-Up Care

**Figure 1:**
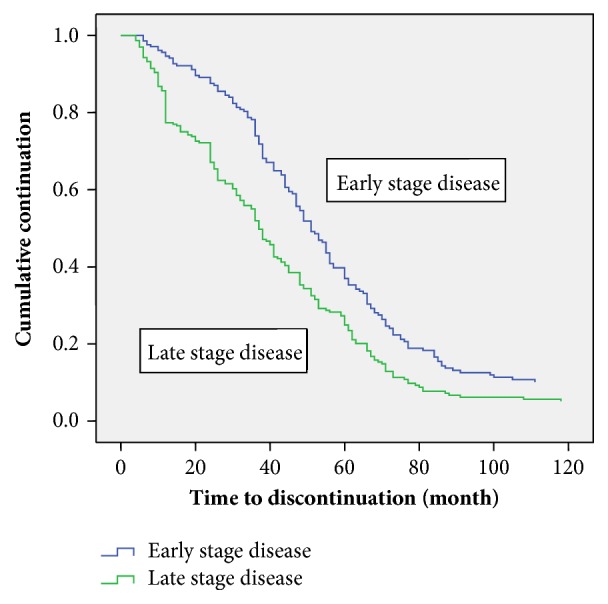
Kaplan Meier discontinuation curve by Manchester stage.

**Figure 2:**
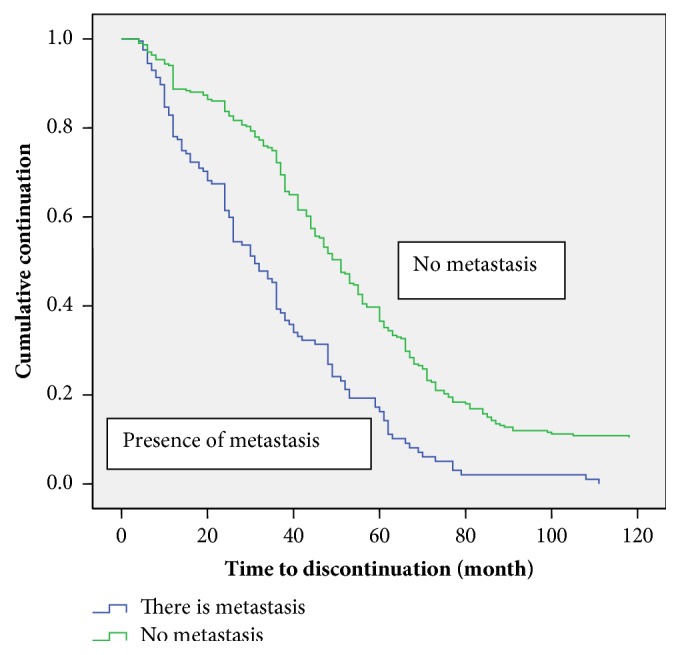
Kaplan-Meier discontinuation curve by metastasis.

**Figure 3:**
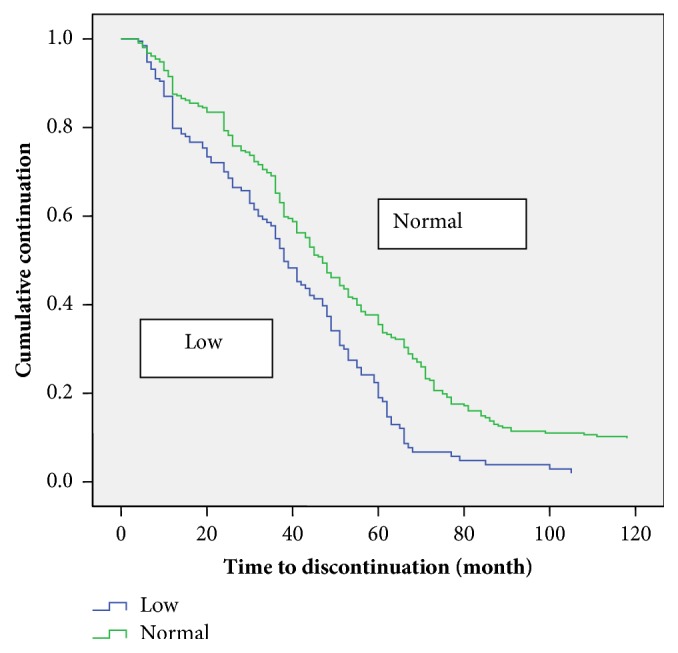
Kaplan-Meier discontinuation curve by PCV status.

Table 3 shows the comparison of the estimation of the median time to discontinue care among the patients. At the bivariate analysis the marital status (p =0.004), duration before diagnosis (p=0.027) and stage of disease before presentation (p<0.001), presence of metastasis (p<0.001), and baseline packed cell volume (p<0.001) were all associated with the median time to discontinuation of care. Those who presented with late-stage disease, had metastasis, and had anaemia were more likely to discontinue follow-up care earlier than others (Figures 1–3).

### 3.4. Predictors of Discontinuation of Breast Cancer Care

The multivariate analysis was done using Cox-proportional hazard model using covariates that showed statistically significant association with loss to follow-up at p <0.1 from Kaplan-Meier method and Log rank test.  Table 4 showed the results of the Cox-proportional hazard model on variables associated with discontinuation of follow-up. Patients with metastasis [HR=1.793; 95% CI = 1.396 - 2.302], anaemia [HR=1.404; 95% CI = 1.120 - 1.760], advanced/late-stage disease [HR=1.310; 95% CI=1.047 - 1.639], and of older age [HR=1.415; 95% CI = 1.044 - 1.917] are significantly more likely to discontinue follow-up early.

## 4. Discussion

The incidence and mortality of breast cancer are falling in the developed world due to early detection and improved treatment methods while, in the developing countries like Nigeria, the opposite is the case. It is an irony of life that cancer in women in the most accessible organ of the body and the one that epitomizes womanhood commonly presents late in Nigeria with advanced stage disease and overwhelming local and systemic metastases.

This study revealed a mean age of patients with breast cancer studied to be 47.69 years with a standard deviation of 10.26 and the peak age of occurrence at 40-49 years. Adesunkanmi in a study at Ile-Ife, Nigeria, found similar demographics of breast cancer patients with a mean age of 48 years, standard deviation of 12.3, and peak age of 40-49 years [3]. The results are also consistent with the findings of Emmanuel and Obaseki from Niger Delta of Nigeria [12]. Bird and colleagues reported a mean age of 48 years from Kenya while Saghir et al. reported a mean age of 47 years from Beirut, Lebanon [13, 14]. However, Massimo reported a mean age of 58.0 from the United States of America [15]. This reiterates the fact that breast cancer patients in this environment are a decade younger than those in Caucasians.

Male breast cancer accounted for 0.8% of cases of breast cancer studied; this differs from previous studies done in Ibadan and Eastern Nigeria by Ogundiran et al. and Dogo et al., where they reported 2.9% and 3.7%, respectively [16, 17]. Our finding is consistent with that of Caucasians where less than 1% are reported [18, 19].

This study showed that 6.5% are nulliparous while the majority 91.4% are either multiparous or grand multiparous. This finding is contrary to the report by other investigators who had reported that the higher the number of full-term pregnancies, the greater the protection from breast cancer. A reduction in the risk of breast cancer by 7% for had been reported for each birth after the first, in the absence of breastfeeding. In addition, women who breastfeed reduce their risk compared to those who do not [11].

The mean duration of symptoms before diagnosis of 11.64 months. This constitutes late presentation. Bray et al. emphasized that prognosis is heavily dependent on stage of disease at presentation [20]. Late presentation with advanced stage of disease (stages III & IV) was seen in 58.2% of the patients studied; unfortunately when compared to earlier studies from Nigeria and sub-Saharan Africa, the outlook remained poor [3, 5, 21–23]. Adesunkanmi, 2006, reported a similar duration of 11.2 months and about 83% of these patients have detectable axillary nodes limited to or beyond ipsilateral axilla by the time of presentation [3]. The late presentation with advanced disease in this study can be explained by a myriad of factors, such as the low and poor level of education and the far distance of the residences of these patients from the referral centre. In this study more than two-thirds (69%) of the patients were not educated beyond secondary level. The low education level may lead to poor understanding of symptoms and compliance with measures at early detection and presentation. In addition almost half of the patients came from far places in Nigeria outside South Western states. The far distances could also be the reason for late presentation at the referral centre.

About 40% of patients in this study had metastasis, which is explainable by the fact that large number of these patients presented late with advanced disease and had palpable axillary nodes within and beyond axilla, a significant proportion (about 40%) presenting for the first time with anaemia. Adisa, 2011, reported a higher figure of 52% with metastasis from Ile-Ife while Bray reported 26% from the United States of America [20–25]. Racial differences in the tumour biology of breast cancer are seen in Nigerians and African Americans compared to Caucasians points to possible differences in the aetiology or in the genetic makeup of the two cases. The tumours in blacks tend to be more aggressive because most tumours diagnosed among blacks are oestrogen receptor negative (ER negative) and ER negative tumours tend to be poorly differentiated with shorter doubling time [25, 26]. ER receptor status could not be determined because it is not routinely tested for in UCH, Ibadan, due to limited resources. A significant proportion of these patients (about 40%) presented with anaemia; this is probably due to the larger number of patients with advanced disease in this study. These patients have bulky disease and high tumour burden in their bodies resulting in recurrent bleeding, excessive increase in metabolic demand, and poor intake due to repeated ill health.

This study found that the predictors of discontinuation among breast cancer patients on follow-up care and therapy include having an advanced stage disease with distant organ metastasis and anaemia. In the studies by Misu, Preethi, and Matthew, from India and Mangardich from South Bronx, the poorest district of the US reported a similar significant association of higher and poor disease burden with loss to follow-up [27, 28]. In the present analysis, patients with metastasis and anaemia were more likely to be lost to follow-up. As the disease progresses, and as the patient grows more dependent, more support is needed to keep up with follow-up care. This assertion is corroborated with the findings by Kaku et al. and Misu and Matthew who observed similar trends among patients with advanced stage cervical cancer in India [26, 29]. Thus patients without metastasis and anaemia and better performance status were more likely and more able to follow through with their care.

This study also found that older patients are more likely to discontinue follow-up care early. This is because older patients may be more dependent on others, may be less assertive due to the realization of inevitability of death at such an advanced age, and would like to avoid further painful treatment. However, younger patients may be more capable and can attend some of the visits on their own without depending on other family members. Fink, Kahn, and Onwusu, in their independent studies, had identified older age as a predictor of discontinuation of care among breast cancer patients on follow-up therapy [30–32]. Overall, follow-up discontinuation among breast cancer patients that are older and anaemic with advanced stage disease and metastasis might be explained by death. These patients have high disease burden that may result into gradual organ and system failure and ultimately death.

The study is limited by its retrospective nature; it was also anticipated that some cases may not contain complete data and therefore was excluded from the study thus lowering the sample size. The study was carried out in a unit of one hospital instead of a multicenter study so generalization of the result of the study is limited. There is lack of funds that could be used to visit/contact each patient lost to follow-up to determine her status and the possible reasons. Despite all the limitations, the study has highlighted an important gap in the management of breast cancer patients which could serve as template in further research and in the generation of hypothesis. This knowledge would also be useful to influence policy towards breast cancer care.

In conclusion patients with breast cancer studied are aged between 27 and 73 years with a modal age of 41-50 years; they have poor educational background and present late to the hospital with advanced disease and have a high follow-up discontinuation rate. Attention to noncompliance with follow-up therapy and supportive care should be given to nonmarried women particularly those with metastasis and anaemia. Such attention might improve breast cancer outcomes in these patients. The hospital management should encourage and support its Hospice and Palliative Care Unit, particularly the community based patients' visits and consultations, to establish unbroken correspondence and links between the hospital and the patients and their relatives/families. This will reduce loss to follow-up. Breast cancer awareness campaigns and education by several groups and organizations in the country on screening methods, risk factors, self-breast examination, and symptoms of breast cancer should be supported and scaled up. This will encourage early presentation with early-stage disease and subsequently adherence with treatment and follow-up. Facilities for screening services in different localities that are affordable and accessible to women at risk of breast cancer should be made accessible to all women to facilitate early detection of breast cancer.

## Figures and Tables

**Table 1:** Sociodemographic characteristics of breast cancer patients seen at UCH, Ibadan,2001-2010.

**Variable**	**Study group**	**Non study group t**		**p value**
No of patients	n=504	n=518		
Age				
Mean	47.69±10.63	48.15±11.44	-0.666	0.506
Sex				
Male	4(0.8%) 5(1.0%)			
Female	500 (99.2%)	513 (99.0%)		
**Variable**	**Frequency (**%**)**			
Age groups distribution.				
≤30	13(2.6%)			
31-40	134(26.6%)			
41-50	172(34.1%)			
51-60	122(24.2%)			
61-70	60(11.9%)			
>70	3(0.6%)			
Educational status				
None	182 (36.1%)			
Below primary	8(1.6%)			
Primary school	41 (8.1%)			
Secondary school	117 (23.2%)			
Tertiary	156 (31.0%)			
Marital status				
Single	23 (4.6%)			
Married	412 (81.7%)			
Divorced	7(1.4%)			
Separated	1 (0.2%)			
Widow	44 (8.7%)			
Unknown	17 (3.4%)			
Parity				
Nulliparity	33 (6.5%)			
Multiparity	230 (45.6%)			
Grand multiparity	231 (45.8%)			
Unknown	10 (2.0%)			
Distance from home town to UCH, Ibadan				
≤ 250KM	274 (54.4%)			
> 250KM	230 (45.6%)			
Duration of symptoms before diagnosis				
≤ 12 months	370 (73.4%)			
> 12 months	134 (26.6%)			
Menopausal Status				
Menopausal	272(54.0%)			
Premenopausal	216(42.9%)			
Unknown	16(3.2%)			

**Table 2:** Clinical characteristics of breast cancer patients seen at UCH, Ibadan, 2001-2010.

**Variable**	**Frequency (**%**)**
Site of disease	
Right	227 (45.0%)
Left	244 (48.4%)
Bilateral	33 (6.5%)
Stage of disease (Manchester staging)	
I	29 (5.8%)
II	177 (35.1%)
III	138 (27.4%)
IV	155 (30.8%)
Unknown	5 (1.0%)
Lymph node status	
No axillary nodes	62 (12.3%)
Nodes limited to ipsilateral axilla	334 (66.3%)
Nodes beyond ipsilateral axilla	84 (16.7%)
Unknown	24 (4.8%)
Histological type	
Invasive ductal carcinoma	443 (87.9%)
Invasive lobular carcinoma	23 (4.6%)
Inflammatory breast cancer	10 (2.0%)
Breast sarcoma	2 (0.2%)
Anaplastic carcinoma	3 (0.6%)
Malignant cystosarcoma phylloides	4 (0.8%)
Mucinous carcinoma	5 (1.0%)
Squamous cell carcinoma	4 (0.8%)
Paget's disease	2 (0.2%)
Others	8 (1.6%)
Histological grade	
I	89 (17.7%)
II	303 (60.1%)
III	84 (16.7%)
Unknown	28 (5.6%)
Surgery	
No surgery	34 (6.7%)
Radical surgery	413 (81.9%)
Breast conservation surgery	57 (11.3%)
Completed chemotherapy	
Yes	498 (98.8%)
No	6 (1.2%)
Completed radiotherapy	
Yes	493 (97.8%)
No	11 (2.2%)
Completed hormonal therapy	
Yes	236 (46.8%)
No	268 (53.2%)
Developed metastasis	
Yes	202 (40.1%)
No	302 (59.9%)
Sites of metastasis	
Lungs	104 (20.6%)
Bone	116 (23.0%)
Liver	40 (7.9%)
Brain	26 (5.2%)
Kidney	1 (0.2%)
Baseline packed cell volume (PCV)	
Low (PCV < 33%)	194 (38.5%)
Normal (PCV ≥ 33%)	310 (61.5%)

**Table 3:** Log rank test estimation of discontinuation of breast cancer patients by clinical features, at UCH, Ibadan, 2001-2010.

Variable	MDT	Log rank	SE[s(t)]	95%CI	P value
	(months)	Chi-square			
Age					
Younger patients	44.0	3.498	2.965	38.18-49.81	0.061
Older patients	44.0		2.325	39.44-48.56	

Educational level					
Primary school and below	41.0	1.053	2.195	36.70-45.30	0.305
Secondary school and above	47.0		2.265	42.60-51.40	

Marital status					
Not married	39.0	8.289	2.315	34.50-43.50	0.004
Married	45.0		1.954	41.20-48.80	

Menopausal status					
Premenopausal	44.0	2.965	3.356	39.40-52.60	0.085
Postmenopausal	45.0		1.876	41.30-48.70	

Duration of symptoms before diagnosis					
Up to 12 months	47.0	4.868	2.233	42.60-51.40	0.027
Above 12 months	37.0		1.914	33.20-40.8	

Distance of home town to UCH to Ibadan					
>250KM	46.0	0.282	2.252	41.60-50.40	0.596
≤ 250KM	41.0		2.772	35.60-46.40	

Stage of disease					
Early stage (I&II)	51.0	19.500	2.416	46.30-55.70	<0.001
Late stage (III&IV)	37.0		1.542	34.00-40.00	

Axillary node status					
No axillary nodes	51.0	1.600	6.717	38.00-64.00	0.198
Presence of axillary nodes	44.0		2.023	40.00-48.00	

Grade of differentiation					
Well differentiated	41.0	1.360	2.729	35.70-46-30	0.244
Moderate/poorly differentiated	45.0		2.334	40.40-49.60	

Metastasis					
Presence of metastasis	31.0	49.761	2.233	26.60-35.40	<0.001
No metastasis	51.0		2.213	46.70-55.30	

Baseline packed cell volume					
Low PCV	38.0	18.775	2.147	33.80-42.20	<0.001
Normal PCV	47.0		2.183	42.70-51.30	

MDT= median discontinuation time; CI= confidence interval; SE= standard error.

**Table 4:** Cox regression analysis of hazard ratios of predictors of discontinuation of breast cancer patients seen at UCH, Ibadan, 2001-2010.

**Variable**	**HR**	**95**%**CI**	**P-value**
Development of metastasis			
There is metastasis	1.793	1.396-2.302	<0.001
No metastasis	1.00		

Baseline packed cell volume (PCV)			
Low (anaemia)	1.404	1.120-1.760	0.005
Normal (no anaemia)	1.00		

Stage at presentation (Manchester stage)			
Early stage (Stage I&II)	1.310	1.047-1.639	0.015
Late stage (Stage III&IV)	1.00		

Duration of symptoms before diagnosis			
≤ 12 months	0.833	0.660-1.051	0.123
> 12 months	1.00		

Menopausal status			
Premenopausal patients	1.063	0.794-1.425	0.680
Postmenopausal patients	1.00		

Age			
Younger patients	1.415	1.044-1.917	0.52
Older patients	1.00		

Marital status			
Not Married	1.256	0.964-1.637	0.091
Married	1.00		

CI = confidence interval; HR = hazard ratio; 1.00 = reference category.

## Data Availability

The data used to support the findings of this study are available from the corresponding author upon request.
